# The evolutionary origin of psychosis

**DOI:** 10.3389/fpsyt.2023.1115929

**Published:** 2023-01-20

**Authors:** Anastasia Levchenko, Fedor Gusev, Evgeny Rogaev

**Affiliations:** ^1^Institute of Translational Biomedicine, Saint Petersburg State University, Saint Petersburg, Russia; ^2^Center for Genetics and Life Sciences, Department of Genetics, Sirius University of Science and Technology, Sochi, Russia; ^3^Vavilov Institute of General Genetics, Russian Academy of Sciences, Moscow, Russia; ^4^Department of Psychiatry, UMass Chan Medical School, Shrewsbury, MA, United States

**Keywords:** default mode network, dreaming, evolution, human accelerated regions, imagination, psychosis, REM sleep, schizophrenia

## Abstract

Imagination, the driving force of creativity, and primary psychosis are human-specific, since we do not observe behaviors in other species that would convincingly suggest they possess the same traits. Both these traits have been linked to the function of the prefrontal cortex, which is the most evolutionarily novel region of the human brain. A number of evolutionarily novel genetic and epigenetic changes that determine the human brain-specific structure and function have been discovered in recent years. Among them are genomic loci subjected to increased rates of single nucleotide substitutions in humans, called human accelerated regions. These mostly regulatory regions are involved in brain development and sometimes contain genetic variants that confer a risk for schizophrenia. On the other hand, neuroimaging data suggest that mind wandering and related phenomena (as a proxy of imagination) are in many ways similar to rapid eye movement dreaming, a function also present in non-human species. Furthermore, both functions are similar to psychosis in several ways: for example, the same brain areas are activated both in dreams and visual hallucinations. In the present Perspective we hypothesize that imagination is an evolutionary adaptation of dreaming, while primary psychosis results from deficient control by higher-order brain areas over imagination. In the light of this, human accelerated regions might be one of the key drivers in evolution of human imagination and the pathogenesis of psychotic disorders.

## 1. Introduction

Primary psychosis could have its roots in the evolution of Homo sapiens (1, 2). It is a pathological state when the affected individual has false perceptual experiences or fixed beliefs that arise in the absence of corresponding external or somatic stimuli and are perceived by the individual as “real,” i.e., there is an impaired reality testing (3). Primary psychosis can take the shape of hallucinations without insight and delusions that are classified as positive symptoms in the clinical picture of schizophrenia (SCZ); psychosis can also be present in other psychiatric disorders, including schizoaffective disorder, bipolar disorder, and major depression (3). Psychotic symptoms are only known in Homo sapiens, whereas other elements of the psychotic disorder SCZ (hyperactivity, deficits in social interaction, and cognitive deficits) can be modeled in other species, for example, in rodents (4).

Imagination, as the driving force of creativity, seems to be exclusively human too and could be linked to psychosis (5–7) through shared evolutionary origins. It is also of notice that one of the main brain structures responsible for creativity, the prefrontal cortex (PFC) (8), is the most evolutionarily novel (9) and is the most affected in a schizophrenic brain (10, 11). Molecular underpinnings of the evolutionarily novel brain structures in Homo sapiens seem to be explained by new patterns of gene expression (12). Thus, the human-specific capacity to imagine things and to create pictures that do not exist in the outside world is a product of activity of neuronal networks regulated by the new patterns of gene expression. Failures in the function of these neuronal networks could be at the origin of psychotic symptoms. These symptoms (hallucinations and delusions) may come as a result of the patient’s incapability to distinguish between the objective reality and pictures created by his own imagination (13). This could signify that mechanisms dedicated to the control of imagination fail in psychotic patients, and this failure probably has its origins in abnormal embryonic development of the brain (14, 15).

## 2. Evolutionary origins of human imagination and psychosis

### 2.1. Human accelerated regions and psychosis

There are several different types of genetic variants present in Homo sapiens, but absent in all other primate, mammalian or vertebrate species, tested so far. The rationale for studying these human-specific variants is that they could be responsible for traits specific to our species, of which the human brain is the most outstanding (9, 12, 16–26). These sequence variants include human-specific accelerated single nucleotide substitutions in evolutionarily conserved regions called human accelerated regions (HARs) (26–32). HARs are short stretches of DNA, about 260 base pairs on average and to 97% non-coding (12). Integration of results from the six original reports (27–32) showed that there are 3171 HARs (23).

Regulatory regions of the genome present a more significant factor than coding genes in the context of the human brain evolution. This observation stems from the 1975’s paper that reported very few differences in amino acid sequences of proteins between humans and chimpanzees despite numerous morphological differences between the two species (33). Later studies showed that human neurodevelopmental genes were subjected to the most prominent positive selection in non-coding, but not in coding sequences (34–37). For example, biological processes of genes with positive selection primarily in non-coding regions and reduced positive selection in coding regions (with the most significant difference between non-coding and coding sequences) were determined using bioinformatic predictions of PANTHER and Gene Ontology classification systems (34). The results indicated “neurogenesis,” “axon guidance,” “regulation of axonogenesis,” “brain development,” “neuron migration,” “positive regulation of neurogenesis,” and “negative regulation of neurogenesis” among the top biological processes. Furthermore, coding sequences in SCZ-associated genes did not undergo accelerated non-synonymous substitutions during human evolution (38). Non-synonymous genetic variants result in different encoded amino acids and may indicate altered functions of proteins. This characterizes neurodevelopmental genes, whereas positive selection occurred in coding sequences of genes with other functions: the immune system and olfaction (34).

There are multiple lines of evidence suggesting the importance of human-specific gene expression regulation for the brain function. An analysis of DNA methylation in the context of human evolution indicated significant CpG hypomethylation of regulatory regions in the human brain compared with non-human primates (39). Studies of the chromatin state (40, 41) identified multiple loci with significant differences across primates, including neuron-specific changes in regulatory regions of SCZ susceptibility genes that are specific to humans. For example, the promoter of contactin 4 (*CNTN4*) that encodes a neural membrane protein is significantly more active in humans (40) and was linked to SCZ *via* both genetics and transcriptomics (42). A chromosome conformation analysis of the developing brain identified a set of human-specific DNA loops between regulatory regions of the genome (43). Overall, gene expression levels are higher in human brains (44), but hundreds of genes also have a human-specific pattern of expression in a brain region-/cell type-specific manner (45). Importantly, genomic loci identified in different assays include a significant proportion of HARs emphasizing their role in human-specific gene expression regulation (39, 43, 45).

In line with these data, a number of genes that are regulated by HARs with empirically confirmed or bioinformatically predicted enhancer activity, are implicated in human-specific development of certain brain areas, including the cortex (9, 23, 26, 46, 47) [also reviewed in Levchenko et al. (12)]. For example, Capra et al. demonstrated that 60% of HARs are enhancers and bind at least one transcription factor different between human and chimpanzee (46). Five of these HARs drove different patterns of gene expression in the central nervous system and limbs (46). A more recent study by Girskis et al. used the improved method “capture massively parallel reporter assay” (caMPRA) to investigate the enhancer activity of HARs (23). The study revealed that 49% of HARs are enhancers active in neural precursor cells and are enriched for binding motifs of transcription factors active during cortical neurogenesis. In addition, 56% of HARs are found in regions of open chromatin in the human (mostly fetal) brain tissue and these results are corroborated by measurements of the epigenetic marks H3K4me1 and H3K27ac indicating active enhancers (23). By integrating these different methods with long-range chromatin interaction data, Girskis et al. (23) refined a set of 63 HARs active in the developing human cortex that may have contributed to the human brain evolution. HARs primarily regulate expression of evolutionarily constrained, loss of function intolerant genes (24).

Multiple studies reported associations of HARs with SCZ. One study showed that the SCZ-associated linkage disequilibrium blocks containing common genetic variants “single nucleotide polymorphisms” (SNPs) associated with SCZ in a genome-wide association study (GWAS) are enriched in genes putatively regulated by HARs (1). These HARs were determined using sequence conservation in primates and the regulated genes are the most conserved in evolution of primates. The candidate SCZ-associated genes regulated by HARs are found in a gene network co-expressed in inhibitory GABAergic interneurons and are involved in regulation of brain development (1). In addition, SCZ-associated HAR-regulated genes are located only inside and at the center of the largest gene network co-expressed in the human PFC, compared with SCZ-associated genes not regulated by HARs and genes regulated by HARs not associated with SCZ (1). Further studies confirmed these results by reporting that genes regulated by HARs and expressed in the brain are enriched among genes discovered in GWASs or genes with rare variants associated with SCZ (9, 48).

Novel open reading frames (nORFs) are short unconventional transcripts transcribed from canonical gene regions, including versions in-frame and not in-frame of coding genes (more rarely, they are intronic and intergenic transcripts) (49). A study used SCZ expression data from the PsychENCODE consortium (50) to select differentially expressed nORFs that were known to be translated into proteins (51). The nORFs and HARs were considered associated if they overlapped or if a HAR was found 100 thousand base pairs (kb) upstream or downstream of a nORF. The study revealed seven differentially expressed nORFs associated with HARs in SCZ patients (51).

A candidate gene-based association study discovered a haplotype in the RNA gene *HAR1A* (one of the few HARs that are not regulatory sequences) associated with auditory hallucinations in patients with psychotic disorders SCZ, schizoaffective disorder, and delusional disorder (52). Interestingly, SCZ alone was not associated with this haplotype. Finally, another association study based on 49 prioritized SNPs located in HARs reported four SNPs (rs3800926, rs3801844, rs764453, and rs77047799) found in four HARs that were significantly associated with SCZ (53). Three of the four SNPs were deemed functional, modifying binding of transcription factors, while two SNPs alter epigenetic marks of active promoters, repressors, or enhancers; the four SNPs also regulate expression of neurodevelopmental genes (53).

### 2.2. Human imagination may have evolved from dreaming

Imagination can be defined as a capacity to create mental images and situations not connected to the current reality (for example, memories are in principle connected to the reality, but not in current time, and imagining a trip to a location never visited before may not have connections with the reality at all). In other words, imagination is the capacity to create a “virtual version of the world.” The term “creativity” has a widespread use in the neuroscientific literature, despite having varying definitions (5, 6, 8, 54, 55). Although it semantically overlaps with the term “imagination,” it bears important differences, as “creativity” often means mental activity leading to discoveries and innovations and often includes the notion of “appropriateness” or “usefulness” [reviewed in Gonen-Yaacovi et al. (8)], which obviously excludes artistic creations.

Imagination seems to be an exclusively human faculty, as we do not observe behaviors in other species that would convincingly suggest they have the same capacity. Not only is imagination the basis of creativity, leading to discoveries and technical advancements of human civilizations (8, 55), it also can be expressed in various forms of art, including visual arts, story-telling, and music. The oldest discovered artistic creations by Homo sapiens date back to 44 thousand years (ky) ago (56). They depict animals and imaginary beings—humans with other animal’s body parts (therianthropes). Our closest known relatives Neanderthals shared ∼92% of HARs with Homo sapiens (12) and they were capable of artistic expression by depicting abstract symbols: the oldest findings date back from approximately 176 (57) to 65 (58) ky ago. Although these Neanderthal creations—annular constructions made of broken stalagmites and a scalariform motif painted on a cave wall—indicate rather limited capacity for artistic expression, symbols in a general sense are elements of a larger imagined picture. The capacity to express abstract symbols might stretch back to 500 ky ago with a zigzag shape drawn by Homo erectus (59), which indicates that capacity for symbolist expression was developing gradually with the appearance of new Hominin species to become mostly developed in Homo sapiens.

One of the possible proxies of human imagination is a deliberate or spontaneous stream of thought while in a relaxed state. The conceptually interrelated activities of mind wandering, spontaneous thoughts, and daydreaming (collectively referred to in this article as MW) are neurobiologically connected to rapid eye movement (REM) sleep that has high chances of dreaming (60). Same brain regions that constitute the default mode network (DMN)—medial PFC, posterior cingulate, and medial temporal regions that include hippocampus—are activated in both functions (60, 61). Researches noticed numerous analogies between brain activity during MW and REM dreaming, but a more pronounced deactivation of lateral PFC regions involved in cognitive control occurs during REM sleep (60). In addition, a disconnection of the medial PFC and temporal regions from posterior cingulate is more pronounced in REM sleep (60, 61). This seems to explain the frequently odd content of dreams stemming from the lack of cognitive control (lateral PFC) and the lack of a constraint by autobiographical memory (hippocampus).

Based on the neuroimaging data, the human imagination may have its evolutionary roots in dreaming and be its evolutionary adaptation. Dreaming that occurs during sleep is an evolutionarily old phenomenon present not only in mammals, including monotremes (62) [the common ancestor with humans existed 163–191 million years ago (63)], but possibly in taxa as distant as cephalopods (the common ancestor with humans existed more than 500 million years ago) (64).

Numerous neurobiological mechanisms have been proposed to explain hallucinations. One of them is aberrant activation of sensory cortices: speech and language processing areas such as the Wernicke’s and Broca’s areas in auditory hallucinations and visual processing areas such as the lingual gyrus and occipital cortex in visual hallucinations (13). The aberrant activation of sensory cortices could modulate the activity of brain areas involved in cognitive control, such as the anterior cingulate and lateral PFC, leading to the perception of the internal activity as externally-generated (13). One of the possible outcomes of this failure in reality and self-monitoring could be the misattribution of the inner speech to an external source in auditory hallucinations (hearing voices). Finally, the neurobiological origin of hallucinations could arise from a failure to suppress irrelevant intrusive memories and a failure in recollection of the context of these memories, which is corroborated by an aberrant activation in hippocampal regions during auditory hallucinations (13).

Thus, REM sleep is similar to MW with reduced connectivity (60, 61), while hallucinations could result from a loss of control by higher-order brain areas (such as lateral PFC) over DMN that is active during MW. In this sense, hallucinations could be akin of “an intrusion of REM sleep into wakefulness” (65). Other neurobiological analogies among these phenomena exist: similar to visual hallucinations, the visual component of REM dreams is associated with activity in sensory cortical areas of the occipital cortex; furthermore, the control by the dorsolateral PFC is reduced in both REM sleep and psychosis, albeit to different degrees (it may be completely silenced in REM sleep, but retains some activity during hallucinations) [reviewed in Waters et al. (65)]. Analogies between imagination and hallucinations similarly include involvement of sensory cortices in both phenomena: the capacity for auditory imagery is dependent on intact auditory areas and the capacity for visual imagery is dependent on intact visual areas of the brain [reviewed in Waters et al. (7)]. Furthermore, evidence indicates that individuals with artistic abilities, such as writers, composers, and painters, may be prone to psychotic manifestations, although full-blown SCZ is not associated with heightened artistic expression [reviewed in Carson (5)]. Nevertheless, polygenic risk scores for SCZ are associated with memberships in artistic societies of actors, dancers, musicians, visual artists, and writers (6).

## 3. Discussion

**FIGURE 1:**
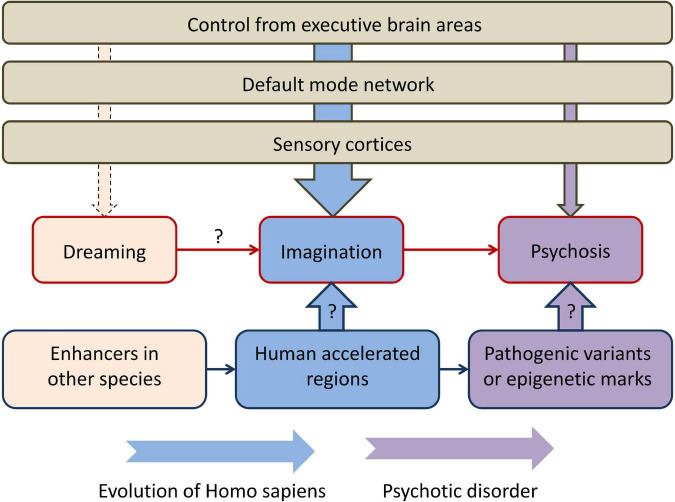
The perspective described in the present article, connecting rapid eye movement dreaming, imagination, and primary psychosis in the context of human brain evolution. “Dreaming” refers to rapid eye movement dreaming and “psychosis” refers to primary psychosis. Higher-order executive brain areas, such as the lateral prefrontal cortex, control the activity of the default mode network and sensory cortices and have different degrees of control over dreaming, imagination, and psychosis. Human accelerated regions could be one of the main genomic components that determine both evolutionary transformations and the pathogenesis of primary psychosis. Arrows with question marks indicate currently limited empirical support.

The perspective described in the present article is summarized in Figure 1. Human imagination could be an evolutionary adaptation that stemmed from the evolutionary old function of dreaming. Overlapping neural networks are implicated in the two functions. From the standpoint of the human genome, HARs could be one of the evolutionarily-novel regulatory sequences that allowed new patterns of gene expression that determined, among other functions, control of the DMN by executive regions (such as lateral PFC). Primary psychosis could have arisen from a failure of the higher-order brain areas to control the DMN activated during normal MW. Psychosis is undoubtedly polygenic (like SCZ and other psychiatric disorders), but pathogenic variants in HARs in particular could be a significant factor in its etiology. An additional source of phenotypic variance could come from altered epigenetic marks in HARs: for example, 48 HARs have increased H3K27ac or H3K4me2 that mark active promoters and enhancers in the human cortex (66). In support of this hypothesis, a number of studies revealed associations between SCZ and genetic variants in HARs. In addition, brain-expressed genes regulated by HARs are significantly more expressed in brain regions of the DMN, where they regulate the formation of synapses and dendrites, compared with other functional brain networks (9). This could indicate HARs are responsible for higher-order cognitive control over human imagination that is a complex function and requires a feedback from higher-order brain areas. The disconnectivity from these brain areas in psychosis is similar to what is occurring during REM sleep.

There are several limitations in the hypothesis linking primary psychosis and HARs. First, because the reported associations are between SCZ and HARs, we cannot be certain the associations are with psychotic symptoms of SCZ and not with cognitive deficits that are also present in more than 85% of patients with this disorder (67). Cognitive deficits in SCZ include not only “neurodegenerative,” but also neurodevelopmental components (14, 68). In fact, other studies reported associations of HARs with autism, a neurodevelopmental disorder (9, 69). Despite this concern, pathogenic genetic variants or epigenetic marks in same HARs could be responsible for both psychosis and cognitive deficits. Furthermore, psychotic symptoms do incorporate some elements of cognitive deficits (70).

The second limitation is the small size of HARs on the genome-wide scale. The entire list of HARs constitutes only about 0.03% of the genome, and this does not account for the fact that the function of HARs was confirmed with multiple methods only for the minority of them. An even smaller proportion of HARs with known functions is relevant to the pathogenesis of psychotic symptoms or the function of the DMN: according to previous studies, as few as 63 HARs determined human brain cortical expansion during evolution (23) and as few as 18 brain-expressed genes regulated by HARs are active in regions of the DMN, where they regulate the formation of synapses and dendrites (9). Nevertheless, because HARs are a product of positive selection (accelerated single nucleotide substitutions) perhaps these sequences do have a tremendous impact on the evolution of the human brain despite the limited space they occupy in the genome. In fact, coding genes constitute only about 1% of the human genome and a few of these genes, like the signaling molecule sonic hedgehog (*SHH*) (71), have a paramount importance for the entire embryonic development.

In the light of these limitations, it is important to state that HARs are most likely not the only evolutionarily novel genome regions that contributed to the emergence of human-specific brain features (16–22). Other types of genetic variants include complex genome rearrangements (72) and a few coding genes with human-specific sequence variants [both reviewed in Levchenko et al. (12)]. Furthermore, human-specific epigenetic modifications are associated with human brain features (39–41, 43). Additional types of evolutionarily novel genomic regions, human gained and human lost enhancers, have different epigenetic profiles in humans compared with chimpanzee, rhesus macaque, and mouse (24, 66). These could also be relevant in the pathogenesis of psychosis, although the current evidence is limited.

Future studies should address the hypothesis described in the present article. For example, genetic variants in HARs could be compared among groups of patients with psychotic disorders categorized according to the severity of psychotic symptoms (i.e., the group with mild psychotic symptoms could be compared with the group with severe psychotic symptoms). Likewise, patients with psychosis could be compared with patients with neurodevelopmental disorders without psychosis, such as intellectual disability. The new results will contribute not only to the search of evolutionary origins of the human brain, but also to the discovery of molecular mechanisms of primary psychosis.

## Data availability statement

The original contributions presented in this study are included in this article/supplementary material, further inquiries can be directed to the corresponding author.

## Author contributions

AL contributed to conception of the mini-review and prepared the first draft. All authors wrote sections of the manuscript, contributed to manuscript revision, and read and approved the submitted version.
